# Quercetin Modulates Ferroptosis via the SIRT1/Nrf−2/HO−1 Pathway and Attenuates Cartilage Destruction in an Osteoarthritis Rat Model

**DOI:** 10.3390/ijms25137461

**Published:** 2024-07-07

**Authors:** Hongri Ruan, Tingting Zhu, Tiantian Wang, Yingchao Guo, Yun Liu, Jiasan Zheng

**Affiliations:** 1 College of Veterinary Medicine, Northeast Agricultural University, Harbin 150030, China; 15776581316@163.com (H.R.); ztt895731877@163.com (T.Z.); 17703649207@163.com (T.W.); cc99@sjtu.edu.cn (Y.G.); 2 College of Animal Science and Veterinary Medicine, Heilongjiang Bayi Agricultural University, Daqing 163000, China

**Keywords:** quercetin, osteoarthritis, ECM, oxidative stress, ferroptosis

## Abstract

Osteoarthritis (OA) is the most common joint disease, causing symptoms such as joint pain, swelling, and deformity, which severely affect patients’ quality of life. Despite advances in medical treatment, OA management remains challenging, necessitating the development of safe and effective drugs. Quercetin (QUE), a natural flavonoid widely found in fruits and vegetables, shows promise due to its broad range of pharmacological effects, particularly in various degenerative diseases. However, its role in preventing OA progression and its underlying mechanisms remain unclear. In this study, we demonstrated that QUE has a protective effect against OA development both in vivo and in vitro, and we elucidated the underlying molecular mechanisms. In vitro, QUE inhibited the expression of IL−1β-induced chondrocyte matrix metalloproteinases (MMP3 and MMP13) and inflammatory mediators such as INOS and COX−2. It also promoted the expression of collagen II, thereby preventing the extracellular matrix (ECM). Mechanistically, QUE exerts its protective effect on chondrocytes by activating the SIRT1/Nrf−2/HO−1 and inhibiting chondrocyte ferroptosis. Similarly, in an OA rat model induced by anterior cruciate ligament transection (ACLT), QUE treatment improved articular cartilage damage, reduced joint pain, and normalized abnormal subchondral bone remodeling. QUE also reduced serum IL−1β, TNF−α, MMP3, CTX−II, and COMP, thereby slowing the progression of OA. QUE exerts chondroprotective effects by inhibiting chondrocyte oxidative damage and ferroptosis through the SIRT1/Nrf−2/HO−1 pathway, effectively alleviating OA progression in rats.

## 1. Introduction

Osteoarthritis (OA) is a common joint disease that affects the entire joint tissue, including cartilage degeneration/loss, subchondral bone remodeling, and synovial fibrosis. Clinically, OA manifests as joint pain, swelling, and limited mobility, and is a leading cause of chronic disability [1]. With the global population aging and obesity rates rising, the incidence of OA is increasing. Statistics indicate that approximately 7% of the world’s population is affected by OA [2]. Currently, no specific therapy exists for OA due to an incomplete understanding of its pathogenesis. Treatment primarily focuses on anti-inflammatory and analgesic drugs, with NSAIDs and acetaminophen being the most commonly used. However, these medications only alleviate the clinical symptoms and offer limited protection against cartilage damage, with their side effects being widely reported [3,4,5]. Despite significant advancements in OA treatments, there remains a pressing need for new therapeutic drugs that are both low in toxicity and have minimal side effects.

Articular cartilage is a connective tissue composed of chondrocytes embedded in an extracellular matrix (ECM) of proteoglycans and collagen. This structure provides load-bearing, shock-absorbing, and lubricating functions. The destruction of the ECM compromises the regulatory effects of cartilage on joint biomechanics, leading to the progression of OA [6,7]. Chondrocytes, the sole cellular components of articular cartilage, are essential for maintaining the balance between the synthesis and degradation of the ECM, thereby preserving the structural integrity of cartilage tissue [8]. Consequently, the impaired activity or death of chondrocytes leads to articular cartilage damage, which is an important factor exacerbating OA. Many studies have indicated that various forms of chondrocyte death are present in the cartilage tissue of OA patients and animal models. Inhibiting chondrocyte death has been shown to improve the prognosis of experimental OA animals [9,10]. Ferroptosis, an iron-dependent programmed cell death first observed in tumor cells in 2012, is triggered by the inactivation of glutathione peroxidase 4 (GPX4) [11]. Recent research has revealed a relationship between OA progression and ferroptosis, with systemic iron overload and increased intracellular iron uptake inducing and exacerbating OA [12,13]. Inhibiting chondrocyte ferroptosis can mitigate ECM degradation, oxidative stress imbalance, and inflammatory damage, thereby improving OA progression [14,15,16,17,18]. Oxidative stress imbalance is widely considered a key factor in ferroptosis. Nrf−2 is a pivotal regulator of antioxidant responses. It plays a crucial role in correcting cellular redox imbalances through its downstream target genes and in regulating the expression of glutathione (GSH) and ferroptosis proteins [19,20]. Several studies have demonstrated that activating Nrf−2 signaling and inhibiting ferroptosis can alleviate OA progression [21,22]. Therefore, regulating Nrf−2 is crucial for improving OA and inhibiting cell ferroptosis.

QUE is a natural flavonoid with promising anti-inflammatory and anti-aging pharmacological activities [23,24]. Multiple recent studies have showcased QUE’s potential to hinder the advancement of OA by regulating inflammatory damage and oxidative stress within articular cartilage [25,26,27,28,29]. Additionally, research by Hu et al. has suggested that QUE may exert chondroprotective effects by influencing synovial macrophage polarization towards the M2 phenotype [30]. Consequently, QUE has emerged as a promising candidate for OA treatment. While prior investigations have demonstrated QUE’s ability to enhance cell ferroptosis sensitivity in conditions such as type 2 diabetes, acute kidney injury, and epilepsy [31,32,33], its role in mitigating OA progression by reducing chondrocyte ferroptosis sensitivity remains unclear. Previous studies have shown that SIRT1 is a crucial target for QUE’s pharmacological activity [14,34]. Building upon this knowledge, we investigated the effects of QUE on chondrocyte extracellular matrix (ECM) degradation, inflammatory mediators, oxidative stress levels, and ferroptosis sensitivity in rat chondrocytes stimulated with IL−1β and in an ACLT-induced rat OA model. The objective was to elucidate the protective effects of QUE on articular cartilage through the SIRT1/Nrf−2/HO−1 pathway and elucidate its underlying mechanisms. We sought to identify the key targets associated with the anti-OA effects of QUE, thereby promoting the development of effective OA therapies.

## 2. Results

### 2.1. Effect of QUE on Chondrocyte Activity

**Figure 1:**
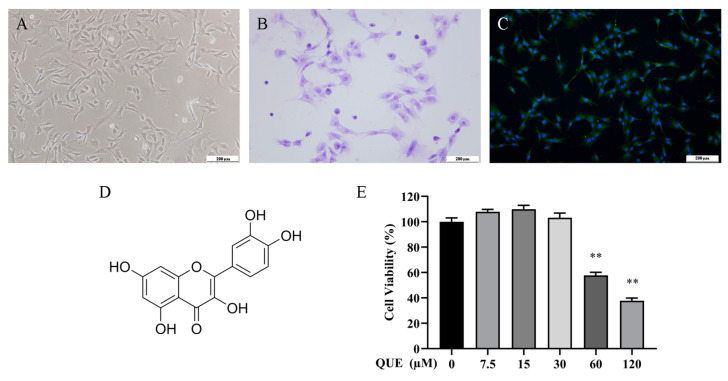
Chondrocyte identification and cell viability detection. (**A**) Representative images of second-generation chondrocytes rat chondrocyte morphology—scale bar, 200 μm. (**B**) Representative image of Toluidine blue staining—scale bar, 200 μm. (**C**) Representative image of collagen II immunofluorescence staining—scale bar, 200 μm. (**D**) Chemical structure of the QUE. (**E**) CCK-8 detected the cell viability of chondrocytes with different concentrations of GC for 24 h. All data are presented as mean ± SD (*n* = 3), ** *p* < 0.01.

P2 chondrocytes exhibited an irregular polygonal shape, as depicted in Figure 1A, and were employed in all subsequent experiments. To validate their identity, toluidine blue staining and type II collagen fluorescence staining were conducted, as illustrated in Figure 1B,C, respectively. Figure 1D illustrates the molecular structure of QUE. Subsequently, we evaluated the impact of QUE on chondrocyte activity (Figure 1E) and observed a significant reduction in chondrocyte activity following treatment with 60 μM QUE. Therefore, we proceeded with further experiments using 7.5 μM, 15 μM, and 30 μM QUE to intervene in chondrocytes, respectively, to investigate the effects of QUE on chondrocytes.

### 2.2. QUE Protects Chondrocytes from IL−1β-Induced Inflammation and Cartilage Degeneration

**Figure 2:**
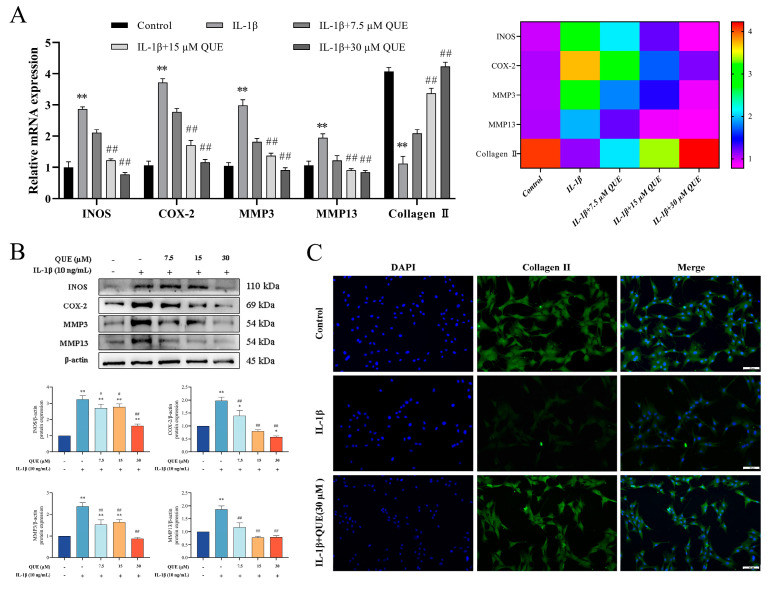
Effect of QUE on the IL−1β-induced inflammatory response of chondrocytes. (**A**) The expression levels of genes associated with chondrocyte inflammation and chondrocyte degeneration were evaluated by qPCR. (**B**) The protein expression of INOS, COX−2, MMP3, and MMP13 were detected by Western blot in the chondrocytes treated with QUE and IL−1β for 24 h. (**C**) Representative images of Collagen II immunofluorescence (green color)—scale bar, 100 μm. All data are presented as mean ± SD (*n* = 3). * *p* < 0.05 and ** *p* < 0.01 compared to the control group; # *p* < 0.05 and ## *p* < 0.01 compared to the IL−1β group.

To assess QUE’s protective effect on chondrocyte ECM degradation, we examined changes in the expression of chondrocyte ECM markers. Fluorescence quantification and protein analysis results (Figure 2A,B) indicated that *INOS*, *COX−2*, *MMP−3*, and *MMP−13* expression levels were significantly elevated in the IL−1β group, whereas QUE dose-dependently inhibited their expression (*p* < 0.01). Furthermore, *Collagen II* mRNA levels were notably reduced in the IL−1β group (*p* < 0.01), which QUE effectively reversed (*p* < 0.01). An immunofluorescence analysis of collagen II also demonstrated QUE’s capacity to enhance its expression (Figure 2C). These findings underscore QUE’s potential chondroprotective role by mitigating IL−1β-induced chondrocyte ECM degradation in a dose-dependent manner, suggesting its therapeutic promise in preventing OA-related cartilage degeneration.

### 2.3. QUE Reduces IL−1β-Induced Ferroptosis and Oxidative Damage in Chondrocytes

**Figure 3:**
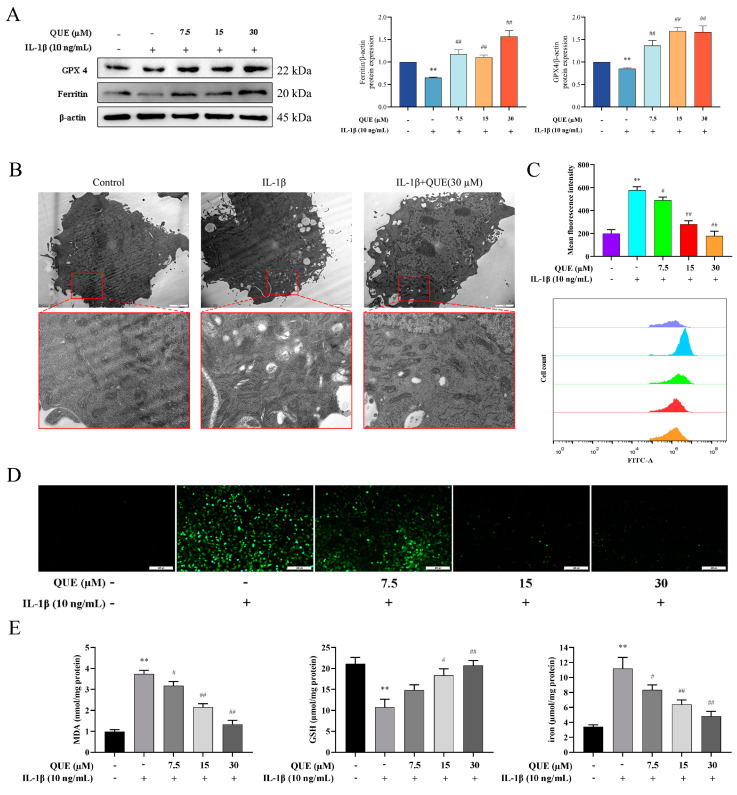
Que improves IL−1β-induced chondrocyte ferroptosis. (**A**) The protein expression of GPX4 and Ferritin were detected by Western blot in the chondrocytes treated with QUE and IL−1β for 24 h. (**B**) Observation of chondrocyte mitochondria with transmission electron microscopy. (**C**) Flow cytometry used to detect ROS levels in chondrocytes. (**D**) Fluorescence microscopy used to observe ROS levels in chondrocytes—scale bar, 200 μm. (**E**) MDA, GSH, and iron concentrations in chondrocytes. All data are presented as mean ± SD (*n* = 3). ** *p* < 0.01 compared to the control group; # *p* < 0.05; ## *p* < 0.01 compared to the IL−1β group.

Next, we examined the expression changes in ferroptosis-related proteins GPX4 and Ferritin, depicted in Figure 3A. IL−1β induced a substantial decrease in GPX4 and Ferritin protein expression levels in chondrocytes (*p* < 0.01). Conversely, following QUE treatment, the expression levels of GPX4 and Ferritin proteins significantly increased in a dose-dependent manner (*p* < 0.01). Furthermore, mitochondrial morphology assessment revealed a reduction in mitochondrial volume and cristae in the IL−1β group. Treatment with 30 μM QUE significantly ameliorated the mitochondrial damage caused by IL−1β (Figure 3B). We also assessed the levels of reactive oxygen species (ROS) in chondrocytes across different groups (Figure 3C,D). IL−1β stimulation significantly increased intracellular ROS levels (*p* < 0.01), while QUE treatment reversed this increase. Notably, treatment with 30 μM QUE had the most pronounced effect in reducing ROS levels (*p* < 0.01). Furthermore, the levels of intracellular malondialdehyde (MDA), glutathione (GSH), and iron followed a similar trend (Figure 3E). In summary, our findings demonstrate that QUE possesses the ability to mitigate IL−1β-induced chondrocyte ferroptosis and oxidative damage.

### 2.4. QUE Improves IL−1β-Induced Chondrocyte Oxidative Damage and Ferroptosis by Regulating the Nrf−2/HO−1 Pathway through SIRT1

**Figure 4:**
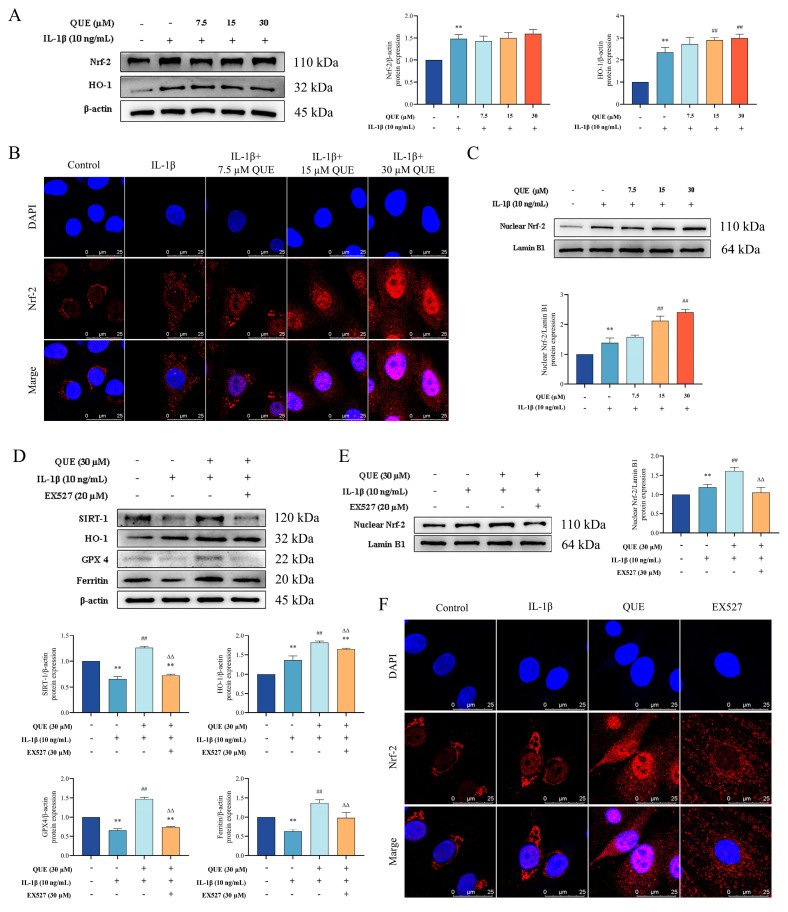
QUE improves IL−1β-induced chondrocyte oxidative damage and ferroptosis through the SIRT1/Nrf−2/HO−1 pathway. (**A**) The protein expression of Nrf−2 and HO−1 were detected by Western blot in the chondrocytes treated with QUE and IL−1β for 24 h. (**B**) Representative images of Nrf−2 immunofluorescence. (**C**) The protein expression of cytoplasm Nrf−2 and nuclear Nrf−2 were detected by Western blot in the chondrocytes treated with QUE and IL−1β for 24 h. (**D**) Western blot image and quantification data for SIRT1, HO−1, GPX4, and Ferritin for each group treated with IL−1β, QUE, and 20 μM EX 527. (**E**) The protein expression of nuclear Nrf−2 was detected by Western blot in the chondrocytes treated with IL−1β, QUE, and 20 μM EX 527. (**F**) Representative images of Nrf−2 immunofluorescence. Data are presented as the means ± SD (*n* = 3). ** *p* < 0.01 compared to the control group; ## *p* < 0.01 compared to the IL−1β group; ∆∆ *p* < 0.01 compared to the QUE group.

The Nrf−2/HO−1 pathway is a critical antioxidant system involved in the pathogenesis of OA and a key regulator of ferroptosis. Given that QUE can mitigate chondrocyte oxidative damage and ferroptosis, we investigated its effects on the Nrf−2/HO−1 pathway. As shown in Figure 4A, IL−1β treatment of chondrocytes significantly increased the protein expression levels of Nrf−2 and HO−1 (*p* < 0.01). Furthermore, QUE treatment further enhanced the protein expression of HO−1 (*p* < 0.01). To analyze the activation status of Nrf−2 more comprehensively, we measured Nrf−2 protein levels in the nucleus and assessed its cellular distribution through immunofluorescence analysis. The results, presented in Figure 4B,C, indicate that IL−1β stimulation significantly upregulated Nrf−2 expression in both compartments (*p* < 0.01). QUE treatment notably increased nuclear Nrf−2 expression in a dose-dependent manner (*p* < 0.01), as confirmed by immunofluorescence, which demonstrated that QUE significantly promoted Nrf−2 nuclear translocation. These findings suggest that IL−1β stimulation activates the Nrf−2/HO−1 pathway in chondrocytes, and QUE treatment can further enhance this pathway, thereby boosting the antioxidant capacity of chondrocytes and preventing oxidative damage.

Recent studies have suggested that QUE modulates the antioxidant capacity of chondrocytes by regulating SIRT1 [35]. Our study found that QUE significantly (*p* < 0.01) increased SIRT1 expression (Figure 4D). To determine whether QUE exerts its protective effects through SIRT1, we used a SIRT1-specific inhibitor (EX527) to explore the underlying mechanism. Compared to the QUE group, the EX527 group exhibited significantly reduced expressions of HO−1, GPX4, ferritin, and nuclear Nrf−2 proteins (*p* < 0.01) (Figure 4D,E). Additionally, the immunofluorescence results showed that EX527 treatment significantly inhibited Nrf−2 nuclear translocation, indicating that SIRT1 inhibition reverses QUE’s effects on enhancing the chondrocyte antioxidant capacity and reducing ferroptosis sensitivity (Figure 4F). Moreover, EX527 inhibits QUE’s protective effects on cartilage ECM (Figure S1). In conclusion, our findings indicate that QUE ameliorates chondrocyte ferroptosis and oxidative damage by modulating the Nrf−2/HO−1 pathway via SIRT1.

### 2.5. Que Improves Joint Pain in OA Rats

**Figure 5:**
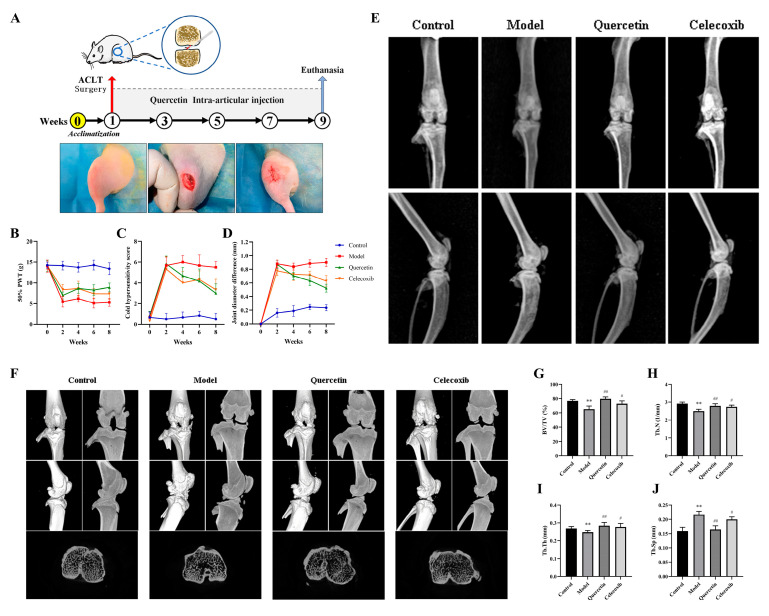
QUE reduces joint pain and joint degeneration in ACLT rats. (**A**) Schematic of the dosing time-course of the rat ACLT-induced knee OA model. (**B**) Mechanical pain domain detection. (**C**) Cold hypersensitivity. (**D**) Knee width measurements. (**E**) Rat knee joint X-ray examination results. (**F**) Micro-CT analysis of proximal tibias from four groups as follows: control, model, quercetin, and celecoxib. (**G**) The bone volume/tissue volume (BV/TV), (**H**) trabecular number (Tb.N), (**I**) trabecular thickness (Tb.Th), and (**J**) trabecular separation (Tb.Sp) were measured to evaluate the microstructure. ** *p* < 0.01 compared to the control group; # *p* < 0.05 and ## *p* < 0.01 compared to the model group.

The experimental procedure and surgical steps for ACLT are depicted in Figure 5A. Eight weeks post-ACLT, joint swelling and pain sensitivity were evaluated through behavioral testing. The results are presented in Figure 5B–D. In the model group, the PWT of rats significantly decreased (*p* < 0.01), and both the cold sensitivity score and joint swelling increased significantly (*p* < 0.01). However, after four weeks of treatment, the QUE and celecoxib groups exhibited significant reductions in joint swelling and cold sensitivity scores compared to the model group (*p* < 0.05), along with significant improvements in pain sensitivity (*p* < 0.01). These results indicate that QUE can ameliorate the joint swelling and pain induced by ACLT in rats. By the eighth week of treatment, the QUE group showed slightly better outcomes in terms of reducing joint swelling and pain than the celecoxib group, although the difference was not statistically significant.

### 2.6. Que Improves Joint Imaging Features in OA Rats

Eight weeks after ACLT, X-ray examinations were performed on the rats in each group. As shown in Figure 5E, the model group exhibited narrowed joint spaces, irregular joint surfaces, and osteophyte formation. The QUE and celecoxib groups, however, maintained normal joint spaces and surface structures with no significant changes. To further evaluate the therapeutic effects of QUE on OA, a micro-CT analysis was performed. The whole-joint 3D reconstruction results are presented in Figure 5F. The model group showed prominent osteophytes around the joint surface and rough joint surfaces. QUE treatment notably reduced osteophyte formation, exhibiting better therapeutic effects compared to the celecoxib group. In addition, four trabecular bone indices were assessed: ACLT surgery significantly decreased BV/TV, Tb.N, and Tb.Th (*p* < 0.01), while increasing Tb.Sp (*p* < 0.01). QUE treatment significantly mitigated these changes (*p* < 0.01), as shown in Figure 5G–J. These findings suggest that QUE can preserve the microarchitecture of subchondral bone and alleviate the progression of ACLT-induced OA in rats.

### 2.7. QUE Improves Cartilage Damage in OA Rats

**Figure 6:**
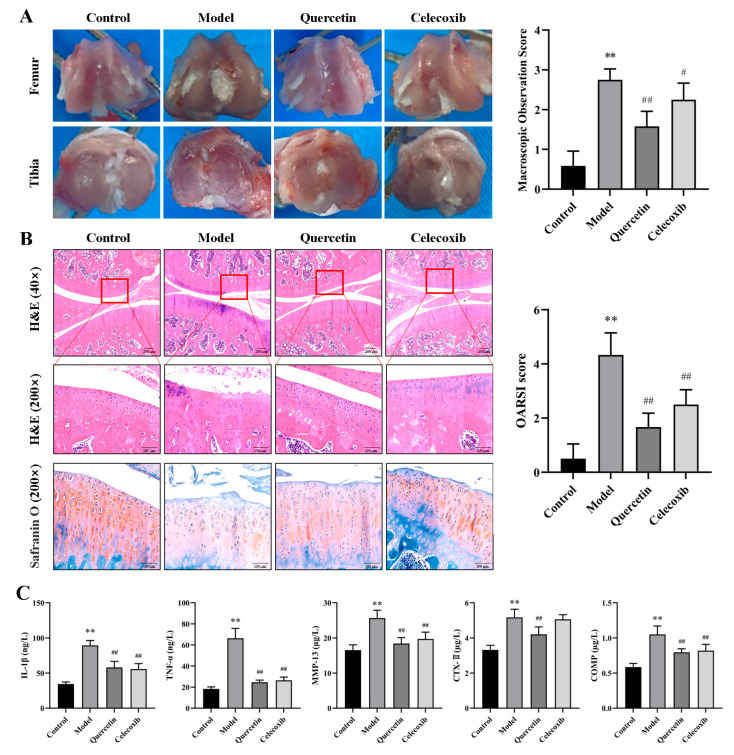
QUE improves the severity of ACLT-induced articular cartilage degeneration in rats. (**A**) Macroscopic observation results and scoring of articular cartilage. (**B**) Representative images of the knee joint stained with hematoxylin-eosin (HE) and OARSI scores after 8 weeks of treatment—scale bar, 200 μm. (**C**) Serum inflammatory factors and cartilage degeneration marker levels. All data are presented as mean ± SD (*n* = 3). ** *p* < 0.01 vs. control group; # *p* < 0.05 and ## *p* < 0.01 vs. model group.

Subsequently, a macroscopic observation of the articular cartilage was conducted to evaluate cartilage damage and assess the protective effect of QUE on rat articular cartilage. The macroscopic observation results and scores of cartilage in OA rats after continuous QUE intervention for 8 weeks are shown in Figure 6A. The articular cartilage of healthy rats is shiny, smooth, and translucent. The model group displayed ulcers on the joint surface, with an uneven and dark red surface, and severe edge wear on the tibia. The QUE group exhibited a rough cartilage surface but no damage. The celecoxib group showed a dull luster with surface ulcers. The results of the joint tissue pathological sections and OARSI scores are presented in Figure 6B. The surface of articular cartilage in healthy rats was not damaged, and the chondrocytes inside were normal in morphology and arranged neatly and orderly. The surface of cartilage in rats in the model group was defective, chondrocytes were hypertrophied, and the ECM staining intensity was reduced. In contrast, the QUE and celecoxib groups showed slightly rough cartilage surfaces, but the cartilage was more complete, and the chondrocytes were arranged more regularly, with significantly lower OARSI scores compared to the model group (*p* < 0.01). The levels of inflammatory factors and cartilage degeneration markers in the serum were also measured. The results demonstrated that QUE significantly ameliorated cartilage damage in OA rats.

### 2.8. QUE Inhibits Cartilage ECM Degradation and Chondrocyte Ferroptosis in OA Rats via Sirt1/Nrf−2/HO−1 Pathway

**Figure 7:**
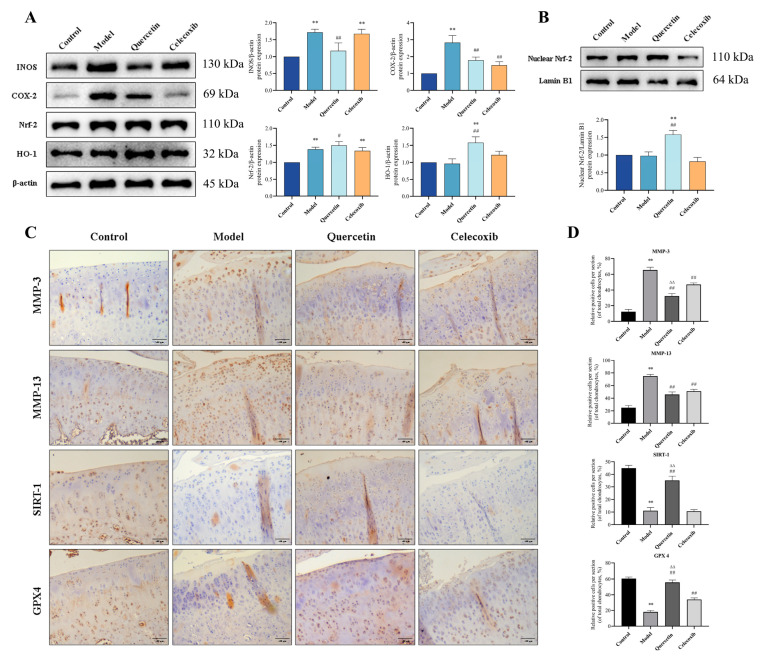
QUE inhibits cartilage ECM degeneration and chondrocyte ferroptosis in OA rats by regulating the SIRT1/Nrf−2/HO−1 pathway. (**A**) The protein expression of INOS, COX−2, Nrf−2, and HO−1 were detected by Western blot in the cartilage tissues of rats in each group. (**B**) The protein expression of nuclear Nrf−2 was detected by Western blot in the cartilage tissues of rats in each group. (**C**) Representative images of immunohistochemical detection of MMP−3, MMP−13, SIRT1, and GPX4 in rat articular cartilage—scale bar, 200 μm. (**D**) Immunohistochemistry quantitative data of MMP−3, MMP−13, SIRT1, and GPX4 in cartilage tissue. ** *p* < 0.01 vs. control group; # *p* < 0.05 and ## *p* < 0.01 vs. model group; ∆∆ *p* < 0.01 compared to the celecoxib group.

To further investigate the protective effects of QUE on cartilage in OA rats, we assessed the protein levels of INOS, COX−2, HO−1, Nrf−2, and nuclear Nrf−2 in the cartilage tissues of each group 8 weeks post-ACLT surgery. The results revealed that QUE treatment significantly reduced (*p* < 0.01) INOS and COX−2 protein levels in cartilage tissue compared to the model group. Additionally, QUE treatment significantly increased (*p* < 0.05) Nrf−2 protein levels and extremely significantly increased (*p* < 0.01) HO−1 and nuclear Nrf−2 protein levels (Figure 7A,B). Immunohistochemical staining and corresponding quantitative analysis (Figure 7C) showed that QUE treatment extremely significantly increased (*p* < 0.01) the rate of SIRT1 and GPX4 positive cells in rat articular cartilage, while it extremely significantly reduced (*p* < 0.01) the rate of MMP−3 and MMP−13 positive cells. These results are consistent with our in vitro experiments, further supporting the protective role of QUE in OA progression.

## 3. Discussion

In recent years, QUE has garnered considerable attention for its diverse pharmacological effects. While previous studies have highlighted QUE’s potential therapeutic benefits in treating arthritis, the precise mechanisms of action remain unclear. Wang et al. demonstrated, in their research, that QUE mitigates oxidative stress damage, thereby attenuating ECM degradation, as well as reducing chondrocyte pyroptosis and apoptosis levels, consequently exerting its therapeutic effects on OA [36]. Wang et al. reported that QUE alleviates articular cartilage degeneration in rats by modulating synovial and systemic inflammatory responses [37]. Similarly, Hu et al. documented QUE’s potent anti-inflammatory effects, altering the inflammatory milieu surrounding articular cartilage and conferring cartilage-protective benefits [30]. In our study, we initially confirmed QUE’s ability to inhibit ECM metabolism and mitigate chondrocyte inflammatory damage both in vivo and in vitro. Additionally, QUE was found to alleviate OA-related pain sensitivity in rats and ameliorate the pathological progression of OA, consistent with previous findings. Furthermore, we observed that QUE reduced chondrocyte sensitivity to ferroptosis and activated the Nrf−2/HO−1 antioxidant pathway. Notably, QUE activation of SIRT1 has been extensively reported in the literature [28]. To elucidate the protective mechanism of QUE on cartilage, we employed a SIRT1-specific inhibitor (EX527). Encouragingly, our results revealed that blocking SIRT1 reversed QUE’s regulatory effects on the Nrf−2/HO−1 pathway and ferroptosis. Consequently, QUE activates the Nrf−2/HO−1 pathway through SIRT1, mitigating chondrocyte oxidative damage and ferroptosis sensitivity, inhibiting ECM degradation and inflammation, and ultimately safeguarding cartilage integrity.

Articular cartilage damage is one of the primary pathological manifestations of OA. The ECM structure of cartilage, comprising collagen and proteoglycan, is crucial for maintaining tissue stability [38]. Matrix metalloproteinases are important indicators of ECM’s metabolic stability, and their increased expression is associated with OA progression [39]. IL−1β, known to induce the expression of various pro-inflammatory and injury-related biomarkers in OA, exacerbates tissue catabolism by upregulating MMPs such as MMP3 and MMP13, thereby creating an inflammatory microenvironment akin to OA [40,41]. In our experiments, QUE counteracted the OA-induced elevation in MMP−13 and MMP−3 expression while augmenting collagen II expression. ACLT surgery has become a widely utilized method for establishing OA models [42,43]. Post-surgery, rats exhibit heightened local joint inflammation, cartilage damage, and pain. Our study observed typical OA features, such as cartilage damage, in the model group via visual and histological examinations, which QUE treatment effectively ameliorated. Radiographic assessments further revealed QUE’s ability to mitigate osteophyte formation and abnormal subchondral bone remodeling. Inflammatory mediators are intimately linked to OA-related pain [44,45]. In our investigation, QUE significantly reduced OA-induced INOS and COX−2 expression in cartilage tissue, along with diminishing serum levels of inflammatory factors IL−1β and TNF−α. Moreover, QUE markedly alleviated the joint pain induced by ACLT in rats, with a therapeutic efficacy comparable to celecoxib. During articular cartilage matrix turnover, degradation products are actively cleaved by enzymes and released into the bloodstream. CTX−II, a collagen II degradation product, and COMP, a non-collagenous articular cartilage protein, are recognized as crucial indicators for assessing knee OA progression [46,47,48,49]. Our study demonstrated QUE’s ability to significantly inhibit ACLT-induced elevations in MMP−13, CTX−II, and COMP serum levels in rats, thereby suppressing ECM degradation in cartilage. Collectively, our in vivo and in vitro findings underscore QUE’s capacity to mitigate the inflammatory damage and cartilage ECM degradation associated with OA, thus playing a protective role in cartilage preservation.

Chondrocytes, the sole cell type in cartilage tissue, play a pivotal role in maintaining the physiological integrity of the cartilage ECM. Preserving the viability of chondrocytes is fundamental for sustaining cartilage’s structure stability, making targeting chondrocyte survival a critical strategy in OA treatment [9,10]. Recent evidence has underscored the intricate interplay between chondrocyte ferroptosis and OA progression. Studies by Yao et al. and Chen et al. revealed that IL−1β and iron overload not only escalate ECM degradation but also precipitate ROS accumulation and upregulate ferroptosis-related proteins, particularly those involving the Nrf−2 antioxidant system [15,50]. Liu et al. further demonstrated that IL−1β induces augmented Fe^2+^ levels in chondrocytes, mitochondrial dysfunction, and intracellular ROS and MDA, as well as other important features of ferroptosis, which can be reduced by activating the Nrf−2 antioxidant system to reduce damage [51]. Our findings corroborate these observations, showing that IL−1β treatment diminishes the expression of GPX4 and Ferritin in chondrocytes, elevates intracellular ROS levels, and induces mitochondrial impairment, indicative of heightened chondrocyte ferroptosis sensitivity. Conversely, QUE effectively counteracts IL−1β-induced chondrocyte damage. Nrf−2, a central player in the cellular antioxidant defense system, is recognized for its role in modulating ferroptosis-related factors and mitigating ferroptosis [52,53]. Zhou et al. demonstrated that reducing chondrocyte ferroptosis sensitivity attenuates OA progression, with Nrf−2 serving as a crucial mediator [54]. In our study, IL−1β treatment activated the overall Nrf−2 antioxidant system in chondrocytes, yet predominantly sequestered Nrf−2 in the cytoplasm. In contrast, QUE treatment significantly augmented Nrf−2 nuclear translocation, enhancing antioxidant system activation. This highlights QUE’s capacity to ameliorate IL−1β-induced chondrocyte damage and ferroptosis sensitivity by bolstering Nrf−2-mediated antioxidant responses.

SIRT1, an NAD-dependent histone deacetylase belonging to the sirtuin family, plays a pivotal role in regulating inflammation and antioxidant defense mechanisms. Emerging evidence suggests that QUE has the ability to activate SIRT1, thereby exerting anti-inflammatory and antioxidant effects [55,56]. Several studies have demonstrated that the Nrf−2/HO−1 antioxidant system can be stimulated through SIRT1 upregulation, resulting in reduced ROS production and the alleviation of cellular damage [14,57]. In our study, we observed that IL−1β downregulates SIRT1 protein expression in chondrocytes, while QUE treatment significantly upregulates SIRT1 protein expression. Notably, the administration of EX527, a SIRT1-specific inhibitor, reversed this effect and attenuated QUE-induced activation of the Nrf−2/HO−1 antioxidant system in chondrocytes. Furthermore, we evaluated the protein expression of ferroptosis-related markers GPX4 and ferritin and found that EX527 not only inhibited SIRT1 protein expression but also counteracted QUE’s suppressive effect on chondrocyte ferroptosis. These findings suggest that QUE’s protective effect on chondrocytes may be mediated by SIRT1 upregulation and the subsequent activation of the Nrf−2/HO−1 antioxidant pathway. Consistent with our in vitro findings, our in vivo experiments demonstrated that QUE activates the SIRT1/Nrf−2/HO−1 pathway in cartilage tissue and enhances GPX4 protein expression, thereby ameliorating cartilage oxidative damage and ferroptosis in OA rats.

While this study confirms that quercetin (QUE) exerts a cartilage-protective role through the SIRT1/Nrf−2/HO−1 pathway, the potential drug toxicity of QUE warrants further investigation. High drug concentrations may not only diminish therapeutic efficacy but could also potentially lead to severe complications. Moreover, the administration of QUE via joint injection in this study aimed to achieve therapeutic concentrations within the joint. However, joint injection can potentially induce tissue damage, necessitating further research into alternative routes of administration to address QUE’s low bioavailability.

## 4. Materials and Methods

### 4.1. Isolation of Chondrocytes

The cartilage tissue was collected from the knee joints of lactating rats (Sprague-Dawley rat (SD rat)) aged 18–21 days, then extracted and digested with 0.25% trypsin (Gibco, Carlsbad, CA, USA) for 1 h. Subsequently, the tissue was incubated with digestion buffer (DMEM/F12 cell culture medium containing 0.2% collagenase II) at 37 °C for 4 h with shaking. After filtration using a Falcon^®^ 70 µm cell strainer (Corning, New York, NY, USA), chondrocytes were collected by centrifugation at 1000 rpm for 5 min and then seeded into 25 cm^2^ cell culture bottles in DMEM/F12 supplemented with 10% fetal calf serum and 1% penicillin—streptomycin (Beyotime, Shanghai, China). The cells were cultured in a 37 °C incubator with 5% carbon dioxide for 3–5 days. Upon reaching approximately 90% confluence, the cells were passaged according to conventional methods. Passage 2 (P2) cells were utilized for experimental purposes in our study. Chondrocytes were characterized through toluidine blue staining and Collagen II immunofluorescence staining before commencing the experiment, and observed using a microscope (Nikon, Tokyo, Japan).

### 4.2. CCK-8 Cell Viability Assay

The chondrocytes were seeded at a density of 1 × 10^4^ cells per well in a 96-well plate and cultured for 18–24 h until fully adherent. Upon reaching confluence, QUE (MedChemExpress, NJ, USA) (7.5, 15, 30, 60, 120 μM) was added to each well for a 24 h treatment period. Subsequently, detection was performed using the CCK-8 assay kit (Beyotime, Shanghai, China), and the OD value of each well at 450 nm was measured using a multifunctional enzyme spectrometer (BioTEK, Winooski, VT, USA).

### 4.3. Detection of Intracellular ROS

The chondrocytes were seeded at a density of 1 × 10^5^ cells per well in a 6-well plate and treated with QUE (7.5, 15, 30 μM) and/or IL−1β for 24 h after reaching confluence. The intracellular ROS level was detected using the DCFH-DA fluorescent probe. Following the addition of DCFH-DA, the cells were incubated for 20–30 min and thoroughly washed with PBS. Subsequently, the intracellular fluorescence of chondrocytes was visualized using fluorescence microscopy (Leica, Weztlar, Germany), and the intracellular ROS flow cytometer (BD, Franklin Lakes, NJ, USA) intensity of each group was quantified using flow cytometry.

### 4.4. MDA, GSH, and Iron Assay

The chondrocytes were seeded at a density of 1 × 10^5^ cells per well in a 6-well plate and cultured for 18–24 h until fully adherent. Upon reaching confluence, QUE (7.5, 15, 30 μM) and/or IL−1β underwent a 24 h treatment period. The concentrations of malondialdehyde (MDA), glutathione (GSH), and iron in the cells were measured using kits provided by Nanjing Jiancheng Biotechnology Co., Ltd (Nanjing, China). Total protein from chondrocytes was extracted via ultrasonic disruption, and the protein concentration of each sample was determined using the BCA detection kit (Beyotime, Shanghai, China). Samples were processed according to the kit instructions. Absorbance readings for each group of cells were taken with a multifunction spectrophotometer (BioTEK, VT, USA). The concentrations of MDA, GSH, and iron ions in each group of cells were then quantified based on the protein concentration.

### 4.5. Observation of Mitochondrial Morphology

Chondrocytes were incubated with IL-1β and/or QUE for 24 h and then fixed with glutaraldehyde for 12–24 h in the dark. Following fixation, the cells were rinsed with 0.1 M PBS buffer solution and then treated with 1% osmium acid solution at 4 °C for 50–60 min in the dark. Subsequently, cells were subjected to gradient dehydration using different concentrations of ethanol (50%, 70%, 90%) for 10 min each. Following this, a mixture of 90% ethanol and 90% acetone in a 1:1 ratio was applied for 10 min, followed by 90% acetone treatment for another 10 min, and finally, three washes with 100% acetone for 10 min each. After dehydration, cells were embedded in epoxy resin using varying acetone-to-epoxy resin ratios (1:1 and 1:2 for 1 h each, and 1:3 overnight). Ultrathin sections, measuring 70–90 nm, were prepared using an ultramicrotome (Leica, Wetzlar, Germany). The samples were then double-stained with uranium and lead, and the sections were stored at room temperature in the dark after staining. Finally, a transmission electron microscope (H-7650, Hitachi Limited, Tokyo, Japan) was used to observe the mitochondrial structure and morphology of the chondrocytes.

### 4.6. RNA Isolation and Reverse Transcription-qPCR

Chondrocytes were treated with QUE (7.5, 15, and 30 μM) and/or IL−1β for 24 h. Using Total RNA Kit (Omega Bio Tek, Doraville, GA, USA) and PrimeScript ™ RT assay kit (Takara, Beijing, China) extracts RNA and synthesizes it into cDNA for subsequent detection. The Cq value of the target gene was determined using a fluorescence quantitative PCR instrument (Roche Light Cycler 480, Basel, Switzerland) with specific primers (Table 1). β-actin served as the internal reference gene. Relative gene expression levels of each target gene were analyzed using the 2^−ΔΔCT^ method.

### 4.7. Immunofluorescence

Initially, cells were seeded in confocal culture dishes and cultured for 18–24 h. Chondrocytes were then treated according to the experimental groups. Cells were fixed with 4% paraformaldehyde for 20–30 min and then washed thoroughly with PBS. Permeabilization was performed using 0.5% Triton X-100 solution for 15 min, followed by blocking with 3% BSA solution to minimize nonspecific binding. Primary antibodies, collagen II antibody (1:200, ABclonal, Shanghai, China), and Nrf-2 antibody (1:200, Proteintech, Wuhan, China), were applied and incubated overnight at 4 °C. Corresponding secondary antibodies, Alexa 488 against collagen II (APExBIO, Houston, TX, USA) and Dylight 649 against Nrf-2 (Abbkine, Wuhan, China), were then added and incubated for 1–1.5 h. DAPI (Beyotime, Shanghai, China) was used for nuclear staining. Finally, fluorescence was observed using a fluorescence microscope (Leica, Weztlar, Germany) or a laser scanning confocal microscope (TCS SP8, Leica, Weztlar, Germany).

### 4.8. OA Induction and Treatment

Thirty male SD rats (10 weeks old, weighing 300–320 g) were obtained from Liaoning Changsheng Experimental Technology Co., Ltd. (Changchun, China). and acclimatized for one week prior to the study. The experiment was divided into four groups (*n* = 8): (1) control group: sham surgery with weekly intra-articular injections of 100 μL sterile PBS buffer containing 0.1% DMSO; (2) model group: ACLT surgery with weekly intra-articular injections of 100 μL sterile PBS buffer containing 0.1% DMSO; (3) QUE group: ACLT surgery with weekly intra-articular injections of 100 μL of 30 μM quercetin (QUE); (4) celecoxib group: positive control with ACLT surgery and oral administration of 2.86 mg/kg/day celecoxib [58]. The surgical procedures were as follows: Rats were anesthetized using inhalation anesthesia (Isoflurane, Ravod Life Sciences, Shenzhen, China) to alleviate pain throughout the surgery. The skin and joint cavity were incised layer by layer, the anterior cruciate ligament was transected under a surgical microscope, and the incision was sutured in layers to prevent tissue adhesion. In the control group, a sham surgery was performed by opening and suturing the joint capsule without ligament damage. One week post-surgery, rats began their respective treatments for 8 weeks. The rats were housed in an environment with a temperature maintained at 27 ± 1 °C, with a relative humidity of 50% ± 12%, and were subjected to a 12 h light/dark exposure cycle. Adequate ventilation was ensured, and the environment underwent regular disinfection. This study adhered strictly to the recommendations outlined in the Guide for the Care and Use of Laboratory Animals of the Ministry of Health, China. All procedures involving animals were approved by the Animal Ethics Committee of Northeast Agricultural University (#NEAUEC2023-03-146-2).

### 4.9. Animal Behavior Tests

During the entire experiment, each group of rats underwent behavioral tests every two weeks, including Knee Width Measurement, the Von–Frey Pain Test, and the Cold Sensitivity Test. The specific test procedures and analysis methods are detailed below.

Knee Width Measurement: To measure the knee joint width, gently stretch the knee joint of the rat to its maximum extension [59]. Measure the maximum diameter of the joint using a vernier caliper, while ensuring that the caliper lightly touches the skin of the rat. Record each measurement result accurately.

Von–Frey Pain Test [60,61]: Place the rat in a plexiglass cage and allow it to acclimate to a quiet environment for 20 min. Once the rat shows no exploratory behavior, use a calibrated Von–Frey mechanical filament to stimulate the center of the right hind paw’s bottom. The sequence of Von–Frey filaments used ranges from 6 to 15 (0.40 g to 15.0 g). Begin the test at the 10th filament (2.0 g) each time, applying vertical force until slight bending occurs, lasting 6–7 s, with a stimulation interval of 3–5 min. Repeat the test three times. Employing the up-and-down method, record a positive “X” when the rat exhibits paw licking or withdrawal, then proceed with the previous filament for further testing. If no positive reaction occurs, record a negative “O” and proceed with the last filament for testing. Perform six consecutive tests; calculate the 50% paw withdrawal threshold (PWT) using the formula 50% PWT(g) = (10[Xf + kδ])/10,000.

Cold Sensitivity Test: Place the rat in a plexiglass cage and allow it to acclimate to a quiet environment for 20 min. Once the rat displays no exploratory behavior, apply a small brush dipped in acetone to the center of the rat’s right hind paw from below. Observe the rat’s behavioral changes over 20 s and record them. Assign a score based on the rat’s behavior records, using a scoring range of 0 to 4 points [62]. Conduct three tests, each with a 20 min interval. Evaluate changes in cold-sensitive response based on the cumulative data from the three tests.

### 4.10. Imaging Examination

X-ray Observation of the Knee Joint: At the 8th week of the experiment, the rat knee joints were examined by X-ray (Long Safe, Northeast Agricultural University). Anteroposterior and lateral radiographs were obtained to assess joint damage.

Micro-CT Observation of the Knee Joint: The fixed rat right knee joint was carefully rinsed with running water. Subsequently, the entire joint tissue sample underwent scanning using the Bruker Micro-CT Skyscan 1276 system (Bruker, Karlsruhe, Germany). Consistent scanning conditions were applied to all experimental samples. Next, the 3D reconstruction software NRecon (1.7.4.2) was employed to reconstruct the scanned original images, and regions of interest (ROI) were selected for analysis. The software CT Analyser (1.18.8.0, Bruker, Karlsruhe, Germany) was utilized for ROI analysis. Finally, quantitative analysis was used for the assessment of BV/TV, Tb.N, Tb.Th, and Tb.Sp.

### 4.11. Macroscopic Observation of Articular Cartilage

Following the experiment, the joint cavity was meticulously opened, and images of the femoral condyle and tibial plateau were captured to assess cartilage damage. Cartilage damage was assessed using a scoring system described by Pelletier [63], which categorizes the extent of articular cartilage damage into grades 0 to 4. All macroscopic observations were conducted by observers blinded to the treatment group allocation.

### 4.12. Histological Analysis

The rat knee joints were fully fixed in 4% formaldehyde solution after all muscle tissues were removed, and then the joint tissues were decalcified with 10% EDTA solution. After decalcification, the knee joints were sectioned and stained with H&E or Safranin O-Fast Green. Finally, the stained sections were evaluated using the OARSI scoring system [64,65].

### 4.13. Immunohistochemical Analysis of Cartilage Tissue

The knee joint tissue sections were initially dewaxed using Pro-Par Clearant (Anatech, NJ, USA), a xylene substitute, followed by rehydration through various concentrations of ethanol. A 10 mM sodium citrate buffer was used to repair antigens. Subsequently, blocking was carried out with goat serum. Primary antibodies targeting MMP-3 (1:200, Proteintech, Wuhan, China), SIRT-1 (1:200, ABclonal, Shanghai, China), MMP-13 (1:200, Proteintech, Wuhan, China), and GPX4 (1:150, ABclonal, Shanghai, China) were applied to the sections, including negative controls, and subsequently incubated in a humidified chamber at 4 °C for 8–12 h. Secondary antibodies corresponding to the primary antibodies were then utilized to bind to the primary antibodies, and the visualization was achieved using diaminobenzidine (DAB). Finally, an optical microscope (Nikon, Tokyo, Japan) was used to capture images and calculate the positive cell rate.

### 4.14. Enzyme-Linked Immunosorbent Assay

At the 8th week of the experiment, 1–1.5 mL of blood was collected from the tail vein of the rats and centrifuged at 4 °C and 3000 rpm for 10 min to separate the serum. The concentrations of IL-1β, TNF-α, MMP-13, CTX-II, and COMP in the serum of each group of rats were determined using ELISA kits. The assays were conducted strictly in accordance with the instructions provided with the ELISA kit (Mlbio, Shanghai, China), with each sample being analyzed in triplicate. The OD values for each well were measured using a multifunctional microplate reader (BioTEK, VT, USA) and the average values were calculated. Finally, the concentrations were determined according to the regression equations of the respective standard curves.

### 4.15. Western Blot Analysis

Chondrocyte proteins were extracted using RIPA lysis buffer (Beyotime, Shanghai, China) containing PMSF (MedChemExpress, Princeton, NJ, USA) and phosphatase inhibitors (MedChemExpress, NJ, USA) at 4 °C for 30 min. The total protein was then collected by centrifugation at 15,000 rpm for 15 min at 4 °C. Additionally, 8 weeks post-surgery, cartilage dissected from the right knee joint of the rats was homogenized with 1.5 mm glass beads in a Mini Bead Beater (BioSpec, Shanghai, China) at 4800 rpm/min for 8–10 min. The tissue homogenate was then subjected to the same protein extraction method, and nuclear protein was extracted using a nuclear protein and cytoplasmic protein extraction kit (Wanleibio, Shenyang, China) according to the manufacturer’s instructions. The protein concentration was determined using the BCA protein analysis kit (Beyotime, Shanghai, China). Thirty micrograms of protein were loaded into each lane, and proteins of different sizes were separated using a 10–12% SDS-PAGE gel and transferred to an NC membrane. All membranes were blocked with 5% bovine serum albumin (BSA, Sigma, Palo Alto, CA, USA) at 37 °C for 1 h. The primary antibodies targeting the proteins of interest were added, including iNOS (1:1500, ABclonal, Shanghai, China), COX-2 (1:1000, ABclonal, Shanghai, China), MMP3 (1:2000), MMP13 (1:2000), GPX4 (1:2000), ferritin (1:2000, ABclonal, Shanghai, China), SIRT-1 (1:2000), Nrf-2 (1:2000, ABclonal, Shanghai, China), HO-1 (1:1000, Abcam, Cambridge, UK), β-actin (1:5000, ABclonal, Shanghai, China), and Lamin B1 (1:1000, ABclonal, Shanghai, China). The NC membrane was incubated with the primary antibodies for 12 h, followed by the removal of the primary antibodies and addition of corresponding secondary antibodies for an additional 0.5–1 h. Subsequently, chemiluminescent solution (Vazyme, Nanjing, China) was used to visualize the proteins, which were observed using a fully automated gel imaging system (Tannon, China). Image J software (1.25a, 1.8.0_112) [66] was utilized for the quantitative analysis of protein expression levels.

### 4.16. Statistical Analysis

Graphs were generated using GraphPad Prism 7 (GraphPad Software, La Jolla, CA, USA), and data are presented as means ± standard deviation. Data analysis was conducted using IBM SPSS Statistics for Windows, Version 22.0 (IBM Corp., Amonk, NY, USA, 2013). Two-tailed Student’s *t*-tests were used for pairwise comparisons between groups, and one-way analysis of variance (ANOVA) was employed for comparisons involving more than two groups, with statistical significance defined as *p* < 0.05. Post hoc tests for multiple comparisons were performed following ANOVA to identify specific group differences.

## 5. Conclusions

In conclusion, our findings demonstrate that QUE exerts a protective effect against OA in rats by reducing OA-induced pain responses, inhibiting cartilage ECM degradation, alleviating inflammatory damage, and mitigating chondrocyte oxidative damage and ferroptosis through activation of the SIRT-1/Nrf-2/HO-1 pathway.

## Figures and Tables

**Table 1:** RT-qPCR detection gene primer sequence.

Gene	Forward Primer (5′−3′)	Reverse Primer (5′−3′)
*MMP3* (NM_133523.3)	TTTGGCCGTCTCTTCCATCC	GCATCGATCTTCTGGACGGT
*MMP13* (NM_133530.1)	TTCTGGTCTTCTGGCACACG	TGGAGCTGCTTGTCCAGGT
*INOS* (NM_012611.4)	AAGAGACGCACAGGCAGAGG	AGCAGGCACACGCAATGATG
*COX−2* (NM_017232.4)	AGAAGCGAGGACCTGGGTTCAC	ACACCTCTCCACCGATGACCTG
*Collagen II* (NM_001414896.1)	ACGAAGCGGCTGGCAACCTCA	CCCTCGGCCCTCATCTCTACATCA
*β-Actin* (NM_031144.3)	TCCCTGGAGAAGAGCTATGA	ATAGAGCCACCAATCCACAC

## Data Availability

The original contributions presented in the study are included in the article/Supplementary Materials; further inquiries can be directed to the corresponding authors.

## Supplementary Materials

The following supporting information can be downloaded at: https://www.mdpi.com/article/10.3390/ijms25137461/s1.
