# Supplementary material for: Quercetin Modulates Ferroptosis via the SIRT1/Nrf−2/HO−1 Pathway and Attenuates Cartilage Destruction in an Osteoarthritis Rat Model
Source: Int J Mol Sci. 2024 Jul 7;25(13):7461. doi: 10.3390/ijms25137461 (PMC11242395; doi:10.3390/ijms25137461)
Supplement: Supplementary file 1 [file ijms-25-07461-s001.zip › Supplementary File 1.pdf]

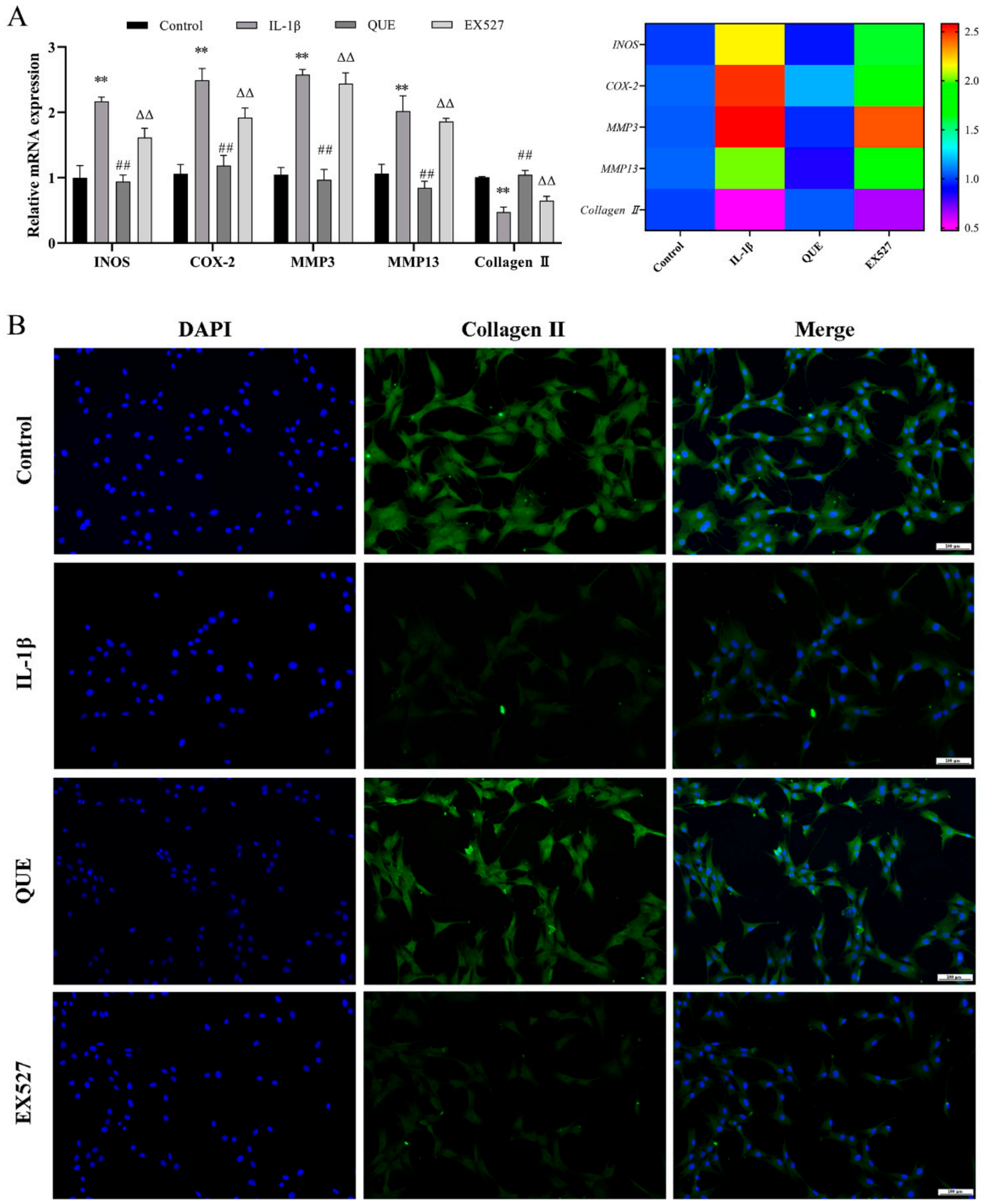

Supplementary Figure S1. QUE improves IL-1β-induced chondrocyte inflammatory response and ECM degradation through the SIRT1/Nrf-2/HO-1 pathway. (A) The expression levels of genes associated with chondrocyte inflammation and chondrocyte de-generation were evaluated by qPCR. (B) Representative images of Collagen II immunofluorescence (green color), scale bar, 100 μm. All data are presented as mean ± SD (n=3). ** $P < 0.01$ compared to the control group; ## $P < 0.01$ compared to the IL-1β group; ΔΔ $P < 0.01$ compared to the QUE group.
